# A Psychological and Linguistic Analysis of “The 2024 State of the Climate Report: Perilous Times on Planet Earth”

**DOI:** 10.1093/biosci/biaf172

**Published:** 2025-11-06

**Authors:** David M Markowitz, Scott Slovic, Paul Slovic

**Affiliations:** Department of Communication, Michigan State University, East Lansing, United States of America; Oregon Research Institute, Springfield, United States of America; Oregon Research Institute, Springfield, United States of America; Department of Psychology, University of Oregon, Eugene, United States of America

**Keywords:** science communication, climate communication, environmental rhetoric, climate change, Alliance of World Scientists

## Abstract

A traditional goal of science and environmental communication, including climate communication, has been to encourage disinterested or uninformed audiences to pay more attention to the world around them and to shift disinterest and apathy toward positive engagement with nature and proenvironment lifestyles. We conducted an empirical investigation of audience responses to key aspects of the world scientists’ “2024 State of the Climate Report: Perilous Times on Planet Earth,” focusing on whether the language of this article manages to sway readers to rethink their attitudes toward climate change. Across many variations, the textual prompts we gave to readers did *not* overwhelmingly move the needle of public attitudes regarding climate change, suggesting that political affiliation and ideologies may be a much stronger indicator of public actions and attitudes than exposure to scientific information. Regarding climate change, we seem to be living in a time of information saturation and ideological entrenchment.

Regarding climate change, we seem to be living in a time of information saturation and ideological entrenchment. The traditional goal of environmental communication, including climate communication, has been to encourage disinterested or uninformed audiences to pay more attention to the world around them and to shift disinterest and apathy toward positive engagement with nature and proenvironment lifestyles. The field of environmental rhetoric developed in the 1990s, through publications such as *Ecospeak: Rhetoric and Environmental Politics in America* (Killingsworth and Palmer 1992) and *Green Culture: Environmental Rhetoric in Contemporary America* (Herndl and Brown 1996), with a strong emphasis on the notion that effective communication strategies might potentially sway readers or viewers toward new ways of thinking about environmental issues, new levels of attentiveness and willingness to participate in prosocial environmental actions.

The efforts of the Alliance of World Scientists (AWS) to contribute to this process of raising public environmental consciousness began with the preparation of the “World Scientists’ Warning to Humanity,” cosigned by approximately 1700 colleagues from around the world (UCS 1992). This document embraces what Killingsworth and Palmer (1992) referred to as “apocalyptic narrative,” along the lines of Rachel Carson's (1962) *Silent Spring*, cautioning readers that “Human beings and the natural world are on a collision course.” Although fundamentally cautionary and informative (in a brief and relatively accessible format), the 1992 warning takes pains not to point fingers and assign blame for the environmental crisis, which includes stratospheric ozone depletion and air pollution, depleted water resources and water pollution, overfishing, the loss of soil productivity, rain forest destruction, and the loss of species. The initial AWS warning addresses the implications of human behavior collectively and seeks to inspire fundamental changes to avoid catastrophic biospheric collapse. This inclusive, global approach has also characterized the many follow-up AWS warning articles, which, as of May 2024, included 49 published articles, a special journal issue, and 73 articles in preparation.

The context and communication strategies of several AWS warnings were also explored in the inaugural issue of *Ecocene: Cappadocia Journal of Environmental Humanities*. While recognizing the importance and admirable goals of the warning articles, the communication specialists quickly recognized many of the shortcomings of these efforts. Slovic (2020), for example, focused on the failure of the warnings to achieve compelling emotional impact for general readers and nonspecialist scientists. Specifically, he writes, “How can science writing about urgent social and environmental issues avoid crushing readers’ attention and potential action through sheer dullness? Unfortunately, I find that the 'World Scientists’ Warning [to Humanity]' delivers a flood of convincing information about the direness of our climate predicament but does so in a way that smothers—that literally *drowns out*—the salience of the warning itself” (p. 46). In addition to the challenge of making a piece of writing about climate change—or other environmental problems—poignant, there is the additional difficulty of nudging a concerned readership to move beyond paralysis and take action of one kind or another.

Against this backdrop, we conducted an empirical analysis of audience responses to key aspects of the world scientists’ “2024 State of the Climate Report: Perilous Times on Planet Earth” (Ripple et al. 2024), focusing on whether traditional climate science communication (AWS language) versus modified versions differentially affects climate attitudes, perceived behavioral control, and behavioral intentions. Since the original AWS warnings more than 30 years ago, the authors have clearly amplified the emotional intensity in comparison with the earlier AWS articles, expressing themselves with the collective first-person pronoun (*we*), repeating the urgent messages (*wrong direction, peril*, and *unprecedented*), and presenting the latest planetary vital signs in a clear and authoritative way that aims to “communicate directly to researchers, policymakers, and the public” (Ripple et al. 2024, p. 1). The article also presents photographs depicting apocalyptic images of floods, fire, and poststorm wreckage. The 14 coauthors are all distinguished climate scientists or environmental communicators, representing countries in many parts of the world. We are unaware of papers that have used the language in AWS reports as stimuli to measure their efficacy in promoting proenvironmental behaviors, despite a wealth of interest in studying how efficacy-related language can amplify feelings and knowledge around climate change in different settings (Ahn et al. 2014, Thaker et al. 2016, Heald 2017, 2019, Bostrom et al. 2019, Loy et al. 2020). Therefore, we addressed an opportunity in the current work to use environmental psychology as a basis to inform how AWS language and other versions based on our experimental paradigm might make people care more about the climate crisis.

At first glance, the AWS report seems to be as concise and clear and forceful an articulation of the current climate crisis as one could imagine. The question is whether this article and communications from the AWS have the potential to change how people think and feel about climate change.

## Evaluating climate messaging efficacy: Theory of planned behavior

Perhaps the most established theoretical framework to evaluate how attitudes toward climate change link to downstream perceptions and behavior is the theory of planned behavior (Ajzen 1988, 1991). The theory has three central components: behavioral intentions based on attitudes, subjective norms, and perceived behavioral control. Behavioral intentions are based on attitudes toward a behavior or target, consisting of evaluative judgments that shape how people think and feel about such targets. Subjective norms are the perceived pressures to engage or not in some action (Ajzen 2020), and perceived behavioral control is akin to one's self-efficacy belief (e.g., one's belief in one's ability to perform some action or behavior; Ajzen 1991). Ultimately, theory of planned behavior research is intended to predict behavior from attitudes, intentions, and beliefs, and it has done so successfully in hundreds of primary studies evaluating how to predict drug use (Armitage et al. 1999), consumer behavior (Ajzen 2015), sex (Bryan et al. 2002), and actions in many other settings. Crucially, at the meta-analytic level, much support has been provided for theory of planned behavior's core ideas, and therefore, study-by-study findings are not elaborated on in the present article (Armitage and Conner 2001, Hagger et al. 2002, Hagger and Chatzisarantis 2009, McDermott et al. 2015, Riebl et al. 2015, Hirschey et al. 2020, Lin and Roberts 2020, Hagger and and Hamilton 2024).

Relevant to the current article, science and climate change communication have been key contexts of study within theory of planned behavior research (Tikir and Lehmann 2011, Clement et al. 2014, Masud et al. 2016, Muñoz et al. 2016, Echegaray and Hansstein 2017, Allen and Marquart-Pyatt 2018, Gkargkavouzi et al. 2019). For example, a recent scoping review evaluated over 120 theory of planned behavior research papers to assess the types of proenvironmental behaviors that are examined in these studies, consider the explanatory power of the theory, and chart pathways forward to study proenvironmentalism in the context of the theory (Yuriev et al. 2020). Among other important findings, the authors concluded that theory of planned behavior research variables such as perceived behavioral control explain a nontrivial amount of variance in predicting attitudes and behaviors, and, crucially, they recommend “scholars should not underestimate the role of organizations in the promotion of sustainable actions” (Yuriev et al. 2020, p. 9). It is important to note that other primary studies have found complementary evidence to support our aims as well. For example, recent work evaluated the impact of risk and efficacy framing on climate change engagement (Chu and Yang 2020). In their experiment, the authors manipulated press stories related to climate change as either emphasizing risks or solutions (e.g., an efficacy frame), observing that “risk framing increased behavioral intention through heightened risk perception” (Chu and Yang 2020, p. 758) and “when communicating impending and concrete risks, stressing the feasibility of action may have stronger motivational potential.” In another study, Morris and colleagues (2019) evaluated how narrative and message structure affected climate change engagement. The authors observed that stories were more effective than informational messages (e.g., those including direct facts) in terms of climate change engagement and encouraging proenvironmental behavior. Altogether, meta-analytic and primary study evidence suggest climate message features (e.g., efficacy, narratives) have the potential to affect how people think and feel about the climate crisis. Building on this empirical foundation, we treat the AWS language as our starting point, using the scientists’ actual words as experimental stimuli to examine how original AWS messaging compares with AWS messaging enhanced with other message features (e.g., efficacy, a news story framing).

We specifically draw on theory of planned behavior research research to measure how the language of AWS communicators can produce different climate attitudes, behavioral intentions, and feelings of perceived behavioral control relative other communication forms. In our first study, we attempted to amplify AWS messaging with additional markers of self-efficacy to improve how people thought and felt about their ability to act against climate change. In study 2, we again altered the form of the AWS messages to also reflect a journalistic narrative. Language clearly matters in climate change communication, changing how people think and feel about the closeness and severity of the crisis (Nerlich et al. 2010, Schuldt et al. 2011, Fløttum 2017). Therefore, we seek to evaluate ways of breaking through to help people feel inclined toward climate action. However, across studies, we observed that it is difficult to move the needle of public attitudes regarding climate change, suggesting that political affiliation and ideologies were a much stronger indicator of public actions and attitudes than exposure to scientific information.

## Study 1: Method

Prior meta-analytic research suggests effect sizes for improving self-efficacy in the context of climate change range of .21 <  *R*^2^ < .31 (Armitage and Conner 2001). Therefore, consistent with our preregistered power analysis (https://aspredicted.org/bwgt-xzpb.pdf), which estimated a medium effect size to achieve 95% power, we needed 210 participants for this study. We nearly doubled this amount to ensure we collected enough participants (final *N* = 403).

Participants from the online research platform, Prolific,were paid $5.00 for their time, and they were, on average, 38.88 years old (standard deviation (*SD*) = 13.03). We requested half of our sample to be self-identifying men (*n* = 197) and half self-identifying women (*n* = 206). We also disaggregated our sample by self-identifying members of the Democratic Party (*n* = 193) and Republican Party (*n* = 193). The remaining participants listed themselves as independents or *other*.

### Procedure

We aimed to evaluate how amplifying climate messaging with efficacy communication might improve people's willingness to engage in climate action, change their climate attitudes, and improve their belief that they can act to improve climate outcomes. Our between-subjects experiment randomly assigned participants to the conclusion of the "2024 State of the Climate Report" (Ripple et al. 2024; we made slight adjustments to the text, such as removing irrelevant references to tables to improve readability for the average participant.), henceforth referred to as our *AWS condition*, or a treatment condition, which we amplified with efficacy messaging using artificial intelligence (AI), henceforth referred to as our *AWS-enhanced condition*. Specifically, we used the chatbot Claude (model 3.5 Sonnet) and provided it with the conclusion of the climate report. Our AI prompt specifically stated, “Make this writing more efficacious. Change as few words as possible, because this might be used as a stimulus in an experiment with the text I just gave you. However, make changes where self-efficacy would increase for people if they read this text.”

All authors on the present article checked the response from the AI and made slight adjustments to the text for clarity. Examples of the textual differences across conditions include, in the AWS condition, “We are currently going in the wrong direction, and our increasing fossil fuel consumption and rising greenhouse gas emissions are driving us toward a climate catastrophe”, and in the AWS-enhanced condition, “While we are currently going in the wrong direction, with increasing fossil fuel consumption and rising greenhouse gas emissions, we have the knowledge and capabilities to prevent a climate catastrophe.” Another example includes, in the AWS condition, “Only through decisive action can we safeguard the natural world, avert profound human suffering, and ensure that future generations inherit the livable world they deserve. The future of humanity hangs in the balance,” and in the AWS enhanced condition, “*Through our collective action*, we can safeguard the natural world, avert profound human suffering, and ensure that future generations inherit the livable world they deserve. The future of humanity *rests in our capable hands*.” The italics are provided for emphasis and did not appear in the study. Full texts with marked changes across conditions are available in the online supplement.

After the participants read the AWS or AWS-enhanced text, they responded to climate-specific questions related to the theory of planned behavior to assess climate attitudes, behavioral intentions, and perceived behavioral control (Masud et al. 2016). We included additional measures to assess behavioral intentions as well (e.g., willingness of participants to donate a portion of their earnings to a proenvironmental charity, willingness to pay a higher price for gasoline if the additional cost was directly used to reduce environmental impacts). The participants then filled out the New Ecological Paradigm (NEP; e.g., a scale to assess how people think and feel about the relationship between humans and the environment; Dunlap et al. 2000), followed by demographics, and exited the survey. All studies reported in this paper were approved by the Michigan State University Institutional Review Board (protocol STUDY00011744).

### Measures

Consistent with prior work (Masud et al. 2016), we evaluated the participants’ climate attitudes (four items; Cronbach's *α* = .82), behavioral intentions (six items; Cronbach's *α* = .89), and perceived behavioral control (three items; Cronbach's *α* = .89) upon reading their randomly assigned environmental message (treatment or control). An example item for climate attitudes is “The environment is in danger because of global climate change,” an example item for behavioral intentions is “I am willing to model proenvironmental actions for my peers,” and an example item for perceived behavioral control is “If everyone took action, we could reduce the impact of climate change.” All items were assessed on a scale of 1 (*strongly disagree*) to 7 (*strongly agree*). Items were randomized to prevent order effects. In studies 1 and 2, we did not include theory of planned behavior research items related to norms because they were not central to our aims.

The participants were told that the researchers would be selecting “a random 10% of participants and asking them to donate some of their earnings to a proenvironmental cause. If selected, how much of your $5.00 payment would you be willing to donate to a proenvironmental cause of our choosing.” A slider from $0.00 to $5.00 asked the participants to indicate their answer. Furthermore, we asked five questions related to proenvironmental sacrifices: the participants’ willingness to pay a higher price for gasoline if the additional cost was directly used to reduce environmental impacts, to support an additional fee on airline tickets if the revenue was used to offset carbon emissions from air travel, to pay more for products with sustainable packaging if it helped reduce waste in landfills, to pay 10% more for groceries if it ensured that sustainable farming practices were used, and to pay more for clothing if it ensured that production was ecofriendly. The participants responded with a binary decision (1, *yes*; 0, *no*).

The participants responded to the 15-item NEP scale, which is a prominent measure of environmental concern. The items were measured on a scale of 1 *(strongly disagree*) to 7 (*strongly disagree*) and include statements such as “We are approaching the limit of the number of people the Earth can support” and “When humans interfere with nature it often produces disastrous consequences.” The items were reliable as a collection (Cronbach's *α* = .88). We used this measure to ensure our randomization was successful (e.g., to ensure environmental concern was evenly distributed across conditions).

### Analytic plan

We used Welch's *t*-tests to examine the relationship between condition and our outcomes of interest. Full statistical outputs for study 1 are reported in the online supplement because of space limitations. The data for the present article are available from the authors upon reasonable request.

## Study 1: Results and discussion

Random assignment between conditions was achieved, as is indicated by consistent rates of environmental concern (*p* = .963), conservatism (*p* = .327), and an even distribution of gender across conditions (*p* = .518). Given the large ideological divide in environmental concern (Johnson and Kennedy 2020), we disaggregate our results by political party.

**Figure 1.**
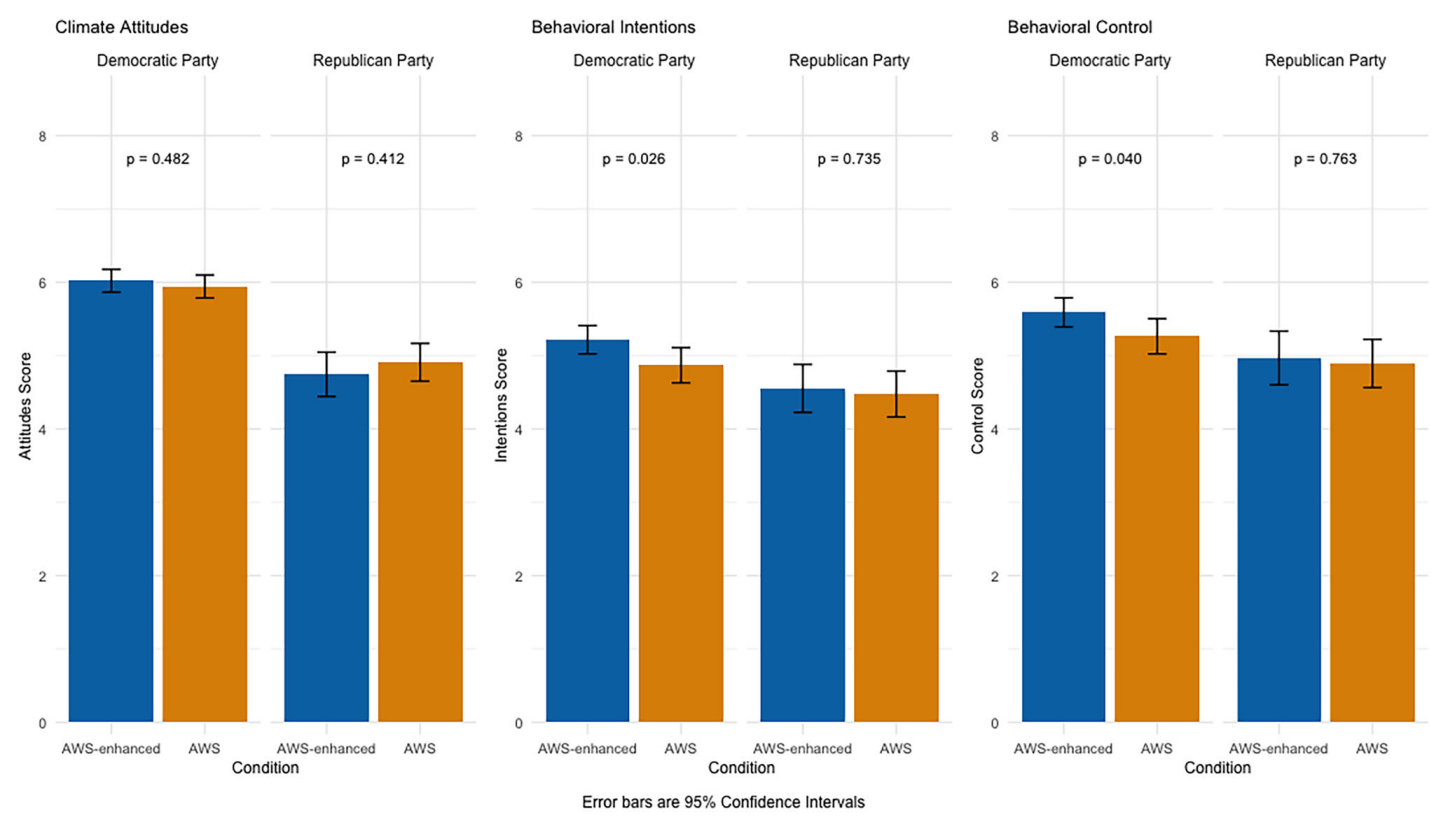
Results for study 1.

The evidence in figure 1 suggests we achieved the goal of improving behavioral intentions (*p* = .026) and perceived behavioral control (*p* = .040) for those in the AWS-enhanced (efficacy) condition relative to the AWS condition, but only among the Democrats. Relationships between condition and various outcome measures were not statistically significant for the Republicans. Regarding additional behavioral intentions, after accounting for political party in a binary logic regression model, only one's willingness to pay additional fees on airline tickets was associated with condition (*B* = -.044, *SE* = 0.22, *z* = -2.04, *p* = .042). Specifically, the participants in the AWS condition were less willing to pay this fee than those in the AWS-enhanced condition.

Together, in study 1, we provide support for the idea that providing efficacious messaging can improve behavioral intentions and perceived behavioral control for Democrats, using the "2024 State of the Climate Report." Although it is suggestive, we aimed to support these data with another investigation to examine how alternative forms of communicating can increase the public's behavioral intentions and sense of perceived behavioral control. We therefore repeated many of the same procedures as study 1 and added two new conditions: On the basis of the conclusion of the "2024 State of the Climate Report," we had AI write a journalistic piece to engage readers and a true control condition in which participants did not read any text. We included the journalistic condition on the basis of prior work that suggests stories can help people be engaged with and care more about climate content (Jones and Peterson 2017).

## Study 2: Method

Given our refined design and additional conditions, we recruited more participants than study 1 (final *N* = 702). Participants from the online research platform, Prolific, were paid $3.50 for their time (this amount of money is slightly lower than in study 1 because of funding constraints.), and they were, on average, 39.68 years old (*SD* = 13.05). Again, we requested half self-identifying men (*n* = 348) and half self-identifying women (*n* = 343) in our sample. We also requested half the sample be self-identifying members of the Democratic Party (*n* = 341) and half of the Republican Party (*n* = 335).

### Procedure

Our procedure in study 2 was identical to study 1 except for the addition of two conditions. The first condition, which we call the *journalistic* condition, was created by soliciting the assistance of Claude (model Sonnet 3.5). This model was provided with the conclusion section of the "2024 State of the Climate Report," and then given the following prompt: “I would like this report to be summarized like a journalist would summarize this for their news readership. Please make this written for a general audience in a journalistic style from a writer at *USA Today*.”

The resulting text, which is in the online supplement, was then edited and reviewed by our authorship team for clarity. In the control condition, the participants did not view any text; instead, they answered the same questions as the participants in the other conditions. In total, we had four conditions: AWS-enhanced (like study 1), AWS (like study 1), journalistic, and the true control condition. The participants answered the same measures in this study as the participants did in study 1 with slight variations as are noted below.

### Measures

We evaluated the participants’ climate attitudes (four items; Cronbach's *α* = .86), behavioral intentions (three items; Cronbach's *α* = .73), and perceived behavioral control (three items; Cronbach's *α* = .89) upon reading their randomly assigned environmental message. We removed three items from the behavioral intentions category because of their repetitiveness. All items were assessed on a scale of 1 (*strongly disagree*) to 7 (*strongly agree*). The items were randomized.

We included the same additional behavioral intentions measures as study 1, except we adjusted the money question to $3.50 on the basis of the participants' payment. We also included a sixth behavioral intention, which asked, “Would you be willing to vote for government officials who are committed to prioritizing climate change mitigation?” The responses were binary (1, *yes*; 0, *no*), and we analyzed them at the item level.

The 15-item NEP measure was statistically reliable as a collection (Cronbach's *α* = .90).

### Analytic Plan

We used Welch's *t*-tests to examine the relationship between condition and our outcomes of interest. The full statistical outputs for study 2 are reported in the online supplement because of space limitations.

## Study 2: Results

**Figure 2.**
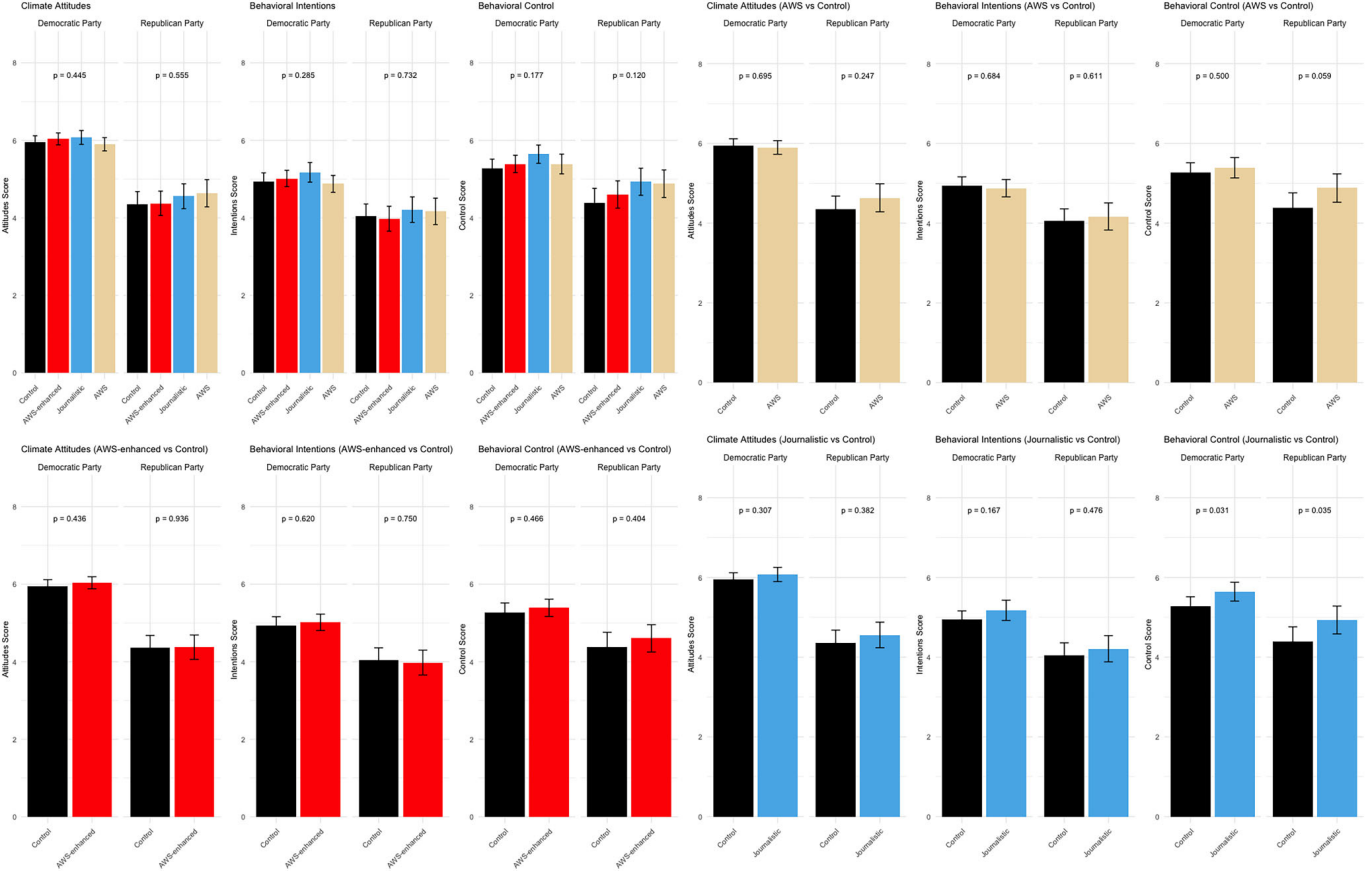
Results for study 2. *Note*. Error bars are 95% Confidence Intervals.

Random assignment was achieved, as is indicated by consistent rates of environmental concern (*p* = .716), conservatism (*p* = .992), and an even distribution of gender across conditions (*p* = .432). Consistent with the prior study, we disaggregate our results by political party. The results at the factor level were not statistically significant (see figure 2 and the online supplement), but our exploratory approach facilitated our interest in probing pairwise comparisons directly.

Figure 2 suggests the most systematic differences across conditions existed for those assigned to read the journalistic story and control. Both the Democratic and the Republican participants reported more perceived behavioral control after reading the journalistic story than after reading the control story (*p*s < .035). There was no effect of condition on additional behavioral intentions.

## General discussion

The results in the present article reveal a challenging reality for climate change messaging. Across both studies, it was difficult to shift attitudes and behavioral intentions regarding climate change, regardless of the communication form provided. Although we observed some modest effects (e.g., when using a journalistic format and efficacy-focused language among the Democrats, the theory of planned behavior research metrics improved), our language-level interventions provided limited evidence to support the idea that amplifying efficacy-based messaging can improve the participants’ reported intentions and perceived control.

Despite these limited findings, we believe this research makes several important contributions. First, we provide evidence that suggests that simply offering scientific information cannot systematically persuade audiences to reconsider their relationship with the environment. Like other work suggests (Chess and Johnson 2007, Hayhoe 2018), information alone, even when presented by authoritative scientific bodies such as the AWS, may be insufficient to overcome ideological divides and entrenchment—at least for highly polarized and partisan issues such as climate change. Although we did not measure entrenchment directly in the current work, this contention is supported by recent evidence suggesting that Democrats in the United States have been more variable over time on their positions related to climate change than Republicans have, but both groups show less variability in their belief that climate change is a major threat over the past half decade (Tyson et al. 2023). Democrats, as we expected, demonstrate higher levels of environmental concern and perceptions of climate change threats than Republicans according to these data. In other words, our evidence and the data from prior work suggest that people are likely stuck in their beliefs regarding climate change according to their political ideology, and language-level changes related to efficacy do little to change how people think and feel about the climate crisis. This is a notable point especially for patterns related to behavioral intentions, values that were near the midpoint for Republicans across conditions. Perhaps the Republican participants may have reached their maximum threshold of information related to climate change regardless of its form and a more useful way to reach these participants (and others) would be to target aspects of social belonging, identity, and trust (e.g., Hamilton et al. 2015, Diehl et al. 2021, Casareale et al. 2023), using the right messengers and messaging format. That is, our work (study 2) suggests that the journalistic style of writing shows some promise in improving theory of planned behavior research variables such as behavioral control, indicating (as other work suggests) that stories are perhaps more important for people than facts in terms of climate change engagement (Morris et al. 2019). This continues to be a fruitful line of future work.

Second, the significance of this work extends beyond academic communities. As climate scientists and environmental organizations invest considerable resources with the hope of crafting warnings and reports (Ripple et al. 2024), understanding the actual impact of these communications (and their associated messengers) on public attitudes and behaviors becomes increasingly crucial. Our approach of using AWS language as experimental stimuli is a novel and valuable methodological contribution to climate communication research. Rather than creating messaging disconnected from real-world climate discourse, we evaluated the effectiveness of actual scientific communication being disseminated to policymakers and the public. This enhances ecological validity while allowing for systematic manipulation of specific linguistic and framing elements. Such methodology could be extended to evaluate other forms of scientific messaging across domains, potentially revealing whether the communication challenges we identified are unique to climate change or are broader obstacles in translating scientific consensus into public action. It is important to note that our use of AI to create efficacy-enhanced messages was a novel methodological contribution, but perhaps the manipulation was not strong enough to systematically change attitudes, beliefs, and intentions. Developing multiple AI-generated stimuli and piloting them prior to experimentation may be helpful to ensure that manipulations are strong enough to facilitate intended effects.

Although the results in the current collection of studies do not demonstrate systematic, foolproof ways to improve climate communication, they also do not demonstrate ways to worsen it. Clearly, more work is needed to find the right messages that can break through and overcome the ideological divides deepening the issues reported in the present article. Science communicators often attempt to provide people with information that can help daily decision-making and improve lives. This should be done in a manner that meets people where they are and communicates the science in an approachable manner (for some examples, see Markowitz 2024). We observed that although our attempts to improve efficacy and environmental behavioral intentions were not universally effective, the current writing and communication styles of AWS may not be having the intended impact, either, as is represented in our results. We encourage scientists of all backgrounds and disciplines to self-reflect on the role that language, storytelling, and format have in communicating climate science. Much more research is required to evaluate the strategies that best communicate the urgency of climate issues accurately, effectively, and in a way that makes people compelled to act (e.g., Chadwick 2015, Bolsen et al. 2019, Goldberg and Gustafson 2025)—and makes them believe they can act too.

We also acknowledge that our interpretation for many null effects in this work stems from the idea of ideological entrenchment. There are other plausible explanations, though, that deserve additional treatment in future research as well. For example, because climate change has historically been a politically polarizing issue (McCright and Dunlap 2010, Antonio and Brulle 2011, Cole et al. 2025), people have been provided information shaping their beliefs and attitudes. Therefore, new information is just the tip of the iceberg of information they have received on the topic, doing little to change or affect how they already think or feel about the crisis. This perspective is supported by information integration theory (Anderson 1971), which suggests people integrate new information with their existing beliefs in a weighted manner. That is, prior knowledge and attitudes heavily influence how new information is processed and incorporated. Testing information integration theory within the scope of our current work is an important and potentially illuminating area of future research.

### Future Directions

The two studies described in the present article were focused on information-based modes of communicating with the public about climate change, using either important passages from the AWS 2024 report or carefully modified versions of these passages (designed to highlight efficacy or to tone down technical scientific language by using a more journalistic voice). With this in mind, future directions for climate communication might transform the information-heavy AWS report into full-fledged narratives, complete with human characters, visualizable events and physical environments, and vivid, accessible representations of the social, economic, and medical consequences of climate change. The journalistic condition therefore could take different forms; rather than reading like a news article, the text could center a character or group of characters, telling their story and their relationship to climate change effects. Unlike the more abstract forms of textual stimuli used in the above-described studies, future studies that use literary or cinematic stories as textual prompts have the potential to encourage emotional reactions through what researchers have called *narrative empathy* (Keen 2007, Zunshine 2010, James 2022), which may lead subjects to break out of the ideological entrenchment that we witnessed in the current studies. Although the results of our current study may be discouraging for climate scientists and others who recognize the urgency of our climate challenges and related ecological crises, we hold out hope that other communication genres and media may still have the potential to jar and excite readers, listeners, and viewers to engage in the decisive action that the world scientists argue is necessary to “safeguard the natural world, avert profound human suffering, and ensure that future generations inherit the livable world they deserve” (Ripple et al., 2024, p. 10). Having more dynamic and interpersonal texts or conversations (instead of static monologues) could be informative as well. Persuasive listening, which is characterized by nonjudgmental and high-quality conversations with others (Grant 2021, Santoro et al. 2025), might be a useful consideration for future research. Finally, identifying causal mechanisms or mediators for studies that measure how language-level changes modify attitudes, behavioral intentions, and perceived behavioral control would be an important area of future work that additional scholarship should explore.

Furthermore, although the original 1992 version of the AWS report was intended to warn all humanity of what lies ahead regarding climate change (UCS 1992), the AWS reports might be written for different constituencies and different sections of the report might be more (or less) relevant for various communities. The following are open empirical questions we hope future scholars will contend with: How does the audience framing of an AWS report affect how people think and feel about climate change? If the report was only targeted toward scientists, or only targeted toward a particular social group, might this have a more profound impact on changing how people think and feel about the crisis? We anticipate generative knowledge to come from these questions and many more.

Finally, it is unclear how deeply the participants read the AWS or AWS-enhanced texts, which presents an opportunity for future research. Some of the participants may have deeply engaged with the writing, whereas others may have had other reading strategies. We expect that deep engagement is a function of interest and acceptance of climate change as a global concern, but this warrants empirical testing.

## Author's note

All authors share lead authorship, and authorship is organized by last name, alphabetically.

## Supplementary Material

biaf172_Supplemental_File

## Author Biography

David M. Markowitz (dmm@msu.edu) is affiliated with Department of Communication, Michigan State University, East Lansing, United state of America. Scott Slovic is affiliated with Oregon Research Institute, Springfield, United state of America. Scott Slovic and Paul Slovic are affiliated with Oregon Research Institute, Springfield, United state of America. Department of Psychology, University of Oregon, Eugene, United state of America.
