# Supplementary material for: A Psychological and Linguistic Analysis of “The 2024 State of the Climate Report: Perilous Times on Planet Earth”
Source: Bioscience. 2025 Nov 6;76(2):171–8. doi: 10.1093/biosci/biaf172 (PMC12856200; doi:10.1093/biosci/biaf172)
Supplement: biaf172_Supplemental_File [file biaf172_supplemental_file.docx]

**Condition Texts**

Study 1 AWS

Despite six Intergovernmental Panel on Climate Change (IPCC) reports, 28 United Nationals Climate Change (COP) meetings, hundreds of other reports, and tens of thousands of scientific papers, the world has made only very minor headway on climate change, in part because of stiff resistance from those benefiting financially from the current fossil-fuel based system. We are currently going in the wrong direction, and our increasing fossil fuel consumption and rising greenhouse gas emissions are driving us toward a climate catastrophe. We fear the danger of climate breakdown. The evidence we observe is both alarming and undeniable, but it is this very shock that drives us to action. We recognize the profound urgency of addressing this global challenge, especially the horrific outlook for the world’s poor. We feel the courage and determination to seek transformative science-based solutions across all aspects of society. Our goal is to provide clear, evidence-based insights that inspire informed and bold responses from citizens to researchers and world leaders.

Rapidly phasing down fossil fuel use should be a top priority. This might be accomplished partly through a sufficiently high global carbon price that could restrain emissions by the wealthy while potentially providing funding for much-needed climate mitigation and adaptation programs. In addition, pricing and reducing methane emissions is critical for effectively mitigating climate change. Methane is a potent greenhouse gas, and unlike carbon dioxide, which persists in the atmosphere for centuries, methane has a relatively short atmospheric lifetime, making reductions impactful in the short term. Drastically cutting methane emissions can slow the near-term rate of global warming, helping to avoid tipping points and extreme climate impacts.

In a world with finite resources, unlimited growth is a perilous illusion. We need bold, transformative change: drastically reducing overconsumption and waste, especially by the affluent, stabilizing and gradually reducing the human population through empowering education and rights for girls and women, reforming food production systems to support more plant-based eating, and adopting an ecological and post-growth economics framework that ensures social justice. Climate change instruction should be integrated into secondary and higher education core curriculums worldwide to raise awareness, improve climate literacy, and empower learners to take action. We also need more immediate efforts to protect, restore, or rewild ecosystems.

The surge in yearly climate disasters shows we are in a major crisis with worse to come if we continue with business as usual. Today, more than ever, our actions matter for the stable climate system that has supported us for thousands of years. Humanity’s future depends on our creativity, moral fiber, and perseverance. We must urgently reduce ecological overshoot and pursue immediate large-scale climate change mitigation and adaptation to limit near-term damage. Only through decisive action can we safeguard the natural world, avert profound human suffering, and ensure that future generations inherit the livable world they deserve. The future of humanity hangs in the balance.

Study 1 AWS-enhanced

Despite six Intergovernmental Panel on Climate Change (IPCC) reports, 28 United Nationals Climate Change (COP) meetings, hundreds of other reports, and tens of thousands of scientific papers, the world has made only very minor headway on climate change, in part because of stiff resistance from those benefiting financially from the current fossil-fuel based system. While we are currently going in the wrong direction, with increasing fossil fuel consumption and rising greenhouse gas emissions, we have the knowledge and capabilities to prevent a climate catastrophe. We recognize the danger of climate breakdown. The evidence we observe is both alarming and undeniable, but it is this very shock that empowers us to action. We know the profound urgency of addressing this global challenge, especially the horrific outlook for the world's poor. We have the courage and determination to implement transformative science-based solutions across all aspects of society. Our goal is to provide clear, evidence-based insights that enable informed and bold responses from citizens to researchers and world leaders.

Rapidly phasing down fossil fuel use should be a top priority. This can be accomplished partly through a sufficiently high global carbon price that could restrain emissions by the wealthy while potentially providing funding for much-needed climate mitigation and adaptation programs. In addition, pricing and reducing methane emissions is critical for effectively mitigating climate change. Methane is a potent greenhouse gas, and unlike carbon dioxide, which persists in the atmosphere for centuries, methane has a relatively short atmospheric lifetime, making reductions immediately beneficial in the short term. By drastically cutting methane emissions we can slow the near-term rate of global warming, helping to avoid tipping points and extreme climate impacts.

In a world with finite resources, unlimited growth is a perilous illusion. We are capable of creating bold, transformative change: drastically reducing overconsumption and waste, especially by the affluent, stabilizing and gradually reducing the human population through empowering education and rights for girls and women, reforming food production systems to support more plant-based eating, and adopting an ecological and post-growth economics framework that ensures social justice. Climate change instruction equips learners in secondary and higher education core curriculums worldwide with the tools to raise awareness, improve climate literacy, and create positive change. We also can implement more immediate efforts to protect, restore, or rewild ecosystems.

The surge in yearly climate disasters shows we are in a major crisis with worse to come if we continue with business as usual. Today, more than ever, our actions create meaningful change for the stable climate system that has supported us for thousands of years. Humanity's future thrives on our creativity, moral fiber, and perseverance. We have the power to reduce ecological overshoot and pursue immediate large-scale climate change mitigation and adaptation to limit near-term damage. Through our collective action, we can safeguard the natural world, avert profound human suffering, and ensure that future generations inherit the livable world they deserve. The future of humanity rests in our capable hands.

Study 2: AWS

Despite six Intergovernmental Panel on Climate Change (IPCC) reports, 28 United Nationals Climate Change (COP) meetings, hundreds of other reports, and tens of thousands of scientific papers, the world has made only very minor headway on climate change, in part because of stiff resistance from those benefiting financially from the current fossil-fuel based system. We are currently going in the wrong direction, and our increasing fossil fuel consumption and rising greenhouse gas emissions are driving us toward a climate catastrophe. We fear the danger of climate breakdown. The evidence we observe is both alarming and undeniable, but it is this very shock that drives us to action. We recognize the profound urgency of addressing this global challenge, especially the horrific outlook for the world’s poor. We feel the courage and determination to seek transformative science-based solutions across all aspects of society. Our goal is to provide clear, evidence-based insights that inspire informed and bold responses from citizens to researchers and world leaders.

Rapidly phasing down fossil fuel use should be a top priority. This might be accomplished partly through a sufficiently high global carbon price that could restrain emissions by the wealthy while potentially providing funding for much-needed climate mitigation and adaptation programs. In addition, pricing and reducing methane emissions is critical for effectively mitigating climate change. Methane is a potent greenhouse gas, and unlike carbon dioxide, which persists in the atmosphere for centuries, methane has a relatively short atmospheric lifetime, making reductions impactful in the short term. Drastically cutting methane emissions can slow the near-term rate of global warming, helping to avoid tipping points and extreme climate impacts.

In a world with finite resources, unlimited growth is a perilous illusion. We need bold, transformative change: drastically reducing overconsumption and waste, especially by the affluent, stabilizing and gradually reducing the human population through empowering education and rights for girls and women, reforming food production systems to support more plant-based eating, and adopting an ecological and post-growth economics framework that ensures social justice. Climate change instruction should be integrated into secondary and higher education core curriculums worldwide to raise awareness, improve climate literacy, and empower learners to take action. We also need more immediate efforts to protect, restore, or rewild ecosystems.

The surge in yearly climate disasters shows we are in a major crisis with worse to come if we continue with business as usual. Today, more than ever, our actions matter for the stable climate system that has supported us for thousands of years. Humanity’s future depends on our creativity, moral fiber, and perseverance. We must urgently reduce ecological overshoot and pursue immediate large-scale climate change mitigation and adaptation to limit near-term damage. Only through decisive action can we safeguard the natural world, avert profound human suffering, and ensure that future generations inherit the livable world they deserve. The future of humanity hangs in the balance.

Study 2: AWS-enhanced

Despite six Intergovernmental Panel on Climate Change (IPCC) reports, 28 United Nationals Climate Change (COP) meetings, hundreds of other reports, and tens of thousands of scientific papers, the world has made only very minor headway on climate change, in part because of stiff resistance from those benefiting financially from the current fossil-fuel based system. While we are currently going in the wrong direction, with increasing fossil fuel consumption and rising greenhouse gas emissions, we have the knowledge and capabilities to prevent a climate catastrophe. We recognize the danger of climate breakdown. The evidence we observe is both alarming and undeniable, but it is this very shock that empowers us to action. We know the profound urgency of addressing this global challenge, especially the horrific outlook for the world's poor. We have the courage and determination to implement transformative science-based solutions across all aspects of society. Our goal is to provide clear, evidence-based insights that enable informed and bold responses from citizens to researchers and world leaders.

Rapidly phasing down fossil fuel use should be a top priority. This can be accomplished partly through a sufficiently high global carbon price that could restrain emissions by the wealthy while potentially providing funding for much-needed climate mitigation and adaptation programs. In addition, pricing and reducing methane emissions is critical for effectively mitigating climate change. Methane is a potent greenhouse gas, and unlike carbon dioxide, which persists in the atmosphere for centuries, methane has a relatively short atmospheric lifetime, making reductions immediately beneficial in the short term. By drastically cutting methane emissions we can slow the near-term rate of global warming, helping to avoid tipping points and extreme climate impacts.

In a world with finite resources, unlimited growth is a perilous illusion. We are capable of creating bold, transformative change: drastically reducing overconsumption and waste, especially by the affluent, stabilizing and gradually reducing the human population through empowering education and rights for girls and women, reforming food production systems to support more plant-based eating, and adopting an ecological and post-growth economics framework that ensures social justice. Climate change instruction equips learners in secondary and higher education core curriculums worldwide with the tools to raise awareness, improve climate literacy, and create positive change. We also can implement more immediate efforts to protect, restore, or rewild ecosystems.

The surge in yearly climate disasters shows we are in a major crisis with worse to come if we continue with business as usual. Today, more than ever, our actions create meaningful change for the stable climate system that has supported us for thousands of years. Humanity's future flows from our creativity, moral fiber, and perseverance. We have the power to reduce ecological overshoot and pursue immediate large-scale climate change mitigation and adaptation to limit near-term damage. Through our collective action, we can safeguard the natural world, avert profound human suffering, and ensure that future generations inherit the livable world they deserve. The future of humanity rests in our capable hands

Study 2: Journalists

In a new report that balances urgency with optimism, leading climate scientists have laid out a clear roadmap for tackling climate change, emphasizing that everyday actions combined with systemic changes can create meaningful impact.

While acknowledging current challenges, including increasing fossil fuel use and rising emissions, the scientists stress that proven solutions already exist. "Humanity's future depends on our creativity, moral fiber, and perseverance," the report states, highlighting numerous ways individuals and communities can make a difference.

The report outlines several immediate actions people can take:

- Shifting to more plant-based meals can significantly reduce individual carbon footprints while supporting sustainable food systems
- Reducing waste and mindful consumption, particularly in wealthy nations, can have immediate positive impacts
- Supporting local initiatives for ecosystem protection and restoration creates tangible environmental benefits
- Engaging with climate education and sharing knowledge helps build community awareness and action
- Joining forces with others to collectively raise awareness and mitigate climate effects

For broader impact, scientists encourage citizens to advocate for key systemic changes like carbon pricing policies and methane emission regulations. These larger-scale solutions become more achievable when supported by informed and engaged communities.

Education emerges as a powerful tool for change, with scientists calling for increased climate literacy in schools. Parents and community members can support this by advocating for climate education in their local school systems and participating in community learning initiatives.

The report specifically highlights how local actions can create ripple effects. When communities invest in ecosystem protection, support sustainable businesses, and implement climate-smart policies, they not only reduce their own environmental impact but also demonstrate successful models for others to follow.

Scientists emphasize that the technology and knowledge needed to address climate change already exists. What's needed now is public engagement and political will. By participating in local government, supporting climate-conscious policies, and making sustainable choices, individuals can help drive the transformation needed.

The climate system that has supported human civilization can still be protected, the scientists note, but it requires action at all levels - from individual choices to international cooperation. They point to encouraging signs of change, including growing renewable energy adoption, increasing climate awareness, and successful community-led environmental initiatives.

The message is clear: while the climate challenge is significant, solutions are within reach. Every action counts, and by working together at local, national, and global levels, communities can create the transformative change needed to ensure a sustainable future. The report concludes that by taking action now, we can help protect both current and future generations while building more resilient and sustainable communities.

The scientists' roadmap shows that by combining individual action with community engagement and policy advocacy, people can play a meaningful role in addressing climate change. This comprehensive approach offers hope while providing practical steps everyone can take to contribute to climate solutions.

**Key Statistical Results for Study 1**

|  |  | AWS enhanced | | AWS | |  |  |  |  |
| --- | --- | --- | --- | --- | --- | --- | --- | --- | --- |
| DV | Party | *M* | *SD* | *M* | *SD* | *t* | *df* | *p* | Cohen's *d* |
| Climate Attitudes | Democratic party | 6.02 | 0.775 | 5.942 | 0.768 | 0.705 | 190.903 | .482 | 0.102 |
| Climate Attitudes | Republican party | 4.745 | 1.466 | 4.909 | 1.27 | -0.822 | 181.501 | .412 | -0.12 |
| Behavioral Intentions | Democratic party | 5.216 | 0.958 | 4.868 | 1.178 | 2.244 | 180.88 | .026 | 0.325 |
| Behavioral Intentions | Republican party | 4.553 | 1.596 | 4.476 | 1.553 | 0.339 | 188.342 | .735 | 0.049 |
| Behavioral Control | Democratic party | 5.588 | 0.991 | 5.263 | 1.179 | 2.072 | 183.473 | .040 | 0.299 |
| Behavioral Control | Republican party | 4.967 | 1.769 | 4.892 | 1.624 | 0.303 | 183.012 | .763 | 0.044 |

**Key Statistical Results for Study 2**

| Comparison | Political Party | DV | Condition 1 | *M* | *SD* | Condition 2 | *M* | *SD* | *t* | *df* | *p* | Cohen's *d* |
| --- | --- | --- | --- | --- | --- | --- | --- | --- | --- | --- | --- | --- |
| AWS-enhanced vs Control | Democratic party | Climate Attitudes | Control | 5.947 | 0.809 | AWS-enhanced | 6.037 | 0.699 | -0.781 | 167.577 | .436 | -0.119 |
| AWS-enhanced vs Control | Republican party | Climate Attitudes | Control | 4.357 | 1.466 | AWS-enhanced | 4.375 | 1.483 | -0.081 | 167.413 | .936 | -0.012 |
| AWS-enhanced vs Control | Democratic party | Behavioral Intentions | Control | 4.94 | 1.046 | AWS-enhanced | 5.016 | 0.957 | -0.497 | 167.994 | .620 | -0.076 |
| AWS-enhanced vs Control | Republican party | Behavioral Intentions | Control | 4.049 | 1.409 | AWS-enhanced | 3.977 | 1.516 | 0.319 | 167.999 | .750 | 0.049 |
| AWS-enhanced vs Control | Democratic party | Behavioral Control | Control | 5.269 | 1.162 | AWS-enhanced | 5.391 | 1.009 | -0.73 | 166.452 | .466 | -0.112 |
| AWS-enhanced vs Control | Republican party | Behavioral Control | Control | 4.386 | 1.702 | AWS-enhanced | 4.602 | 1.663 | -0.836 | 166.53 | .404 | -0.128 |
| AWS vs Control | Democratic party | Climate Attitudes | Control | 5.947 | 0.809 | AWS | 5.899 | 0.812 | 0.393 | 175.996 | .695 | 0.059 |
| AWS vs Control | Republican party | Climate Attitudes | Control | 4.357 | 1.466 | AWS | 4.635 | 1.628 | -1.163 | 164.222 | .247 | -0.180 |
| AWS vs Control | Democratic party | Behavioral Intentions | Control | 4.94 | 1.046 | AWS | 4.876 | 1.036 | 0.408 | 175.984 | .684 | 0.061 |
| AWS vs Control | Republican party | Behavioral Intentions | Control | 4.049 | 1.409 | AWS | 4.167 | 1.59 | -0.509 | 165.123 | .611 | -0.078 |
| AWS vs Control | Democratic party | Behavioral Control | Control | 5.269 | 1.162 | AWS | 5.39 | 1.209 | -0.676 | 174.859 | .500 | -0.102 |
| AWS vs Control | Republican party | Behavioral Control | Control | 4.386 | 1.702 | AWS | 4.88 | 1.661 | -1.901 | 165.149 | .059 | -0.294 |
| Journalists vs Control | Democratic party | Climate Attitudes | Control | 5.947 | 0.809 | Journalists | 6.074 | 0.811 | -1.024 | 166.397 | .307 | -0.157 |
| Journalists vs Control | Republican party | Climate Attitudes | Control | 4.357 | 1.466 | Journalists | 4.557 | 1.433 | -0.876 | 158.963 | .382 | -0.138 |
| Journalists vs Control | Democratic party | Behavioral Intentions | Control | 4.94 | 1.046 | Journalists | 5.175 | 1.156 | -1.388 | 163.616 | .167 | -0.213 |
| Journalists vs Control | Republican party | Behavioral Intentions | Control | 4.049 | 1.409 | Journalists | 4.211 | 1.472 | -0.714 | 157.956 | .476 | -0.113 |
| Journalists vs Control | Democratic party | Behavioral Control | Control | 5.269 | 1.162 | Journalists | 5.642 | 1.073 | -2.178 | 167.987 | .031 | -0.333 |
| Journalists vs Control | Republican party | Behavioral Control | Control | 4.386 | 1.702 | Journalists | 4.932 | 1.563 | -2.123 | 158.643 | .035 | -0.334 |
